# Altered DNA methylation at age-associated CpG sites in children with growth disorders: impact on age estimation?

**DOI:** 10.1007/s00414-022-02826-w

**Published:** 2022-05-12

**Authors:** F. Mayer, J. Becker, C. Reinauer, P. Böhme, S. B. Eickhoff, B. Koop, T. Gündüz, J. Blum, W. Wagner, S. Ritz-Timme

**Affiliations:** 1 Institute of Legal Medicine, University Hospital Düsseldorf, 40225 Düsseldorf, Germany; 2 Department of General Paediatrics, University Hospital Düsseldorf, 40225 Düsseldorf, Germany; 3 Institute for Systems Neuroscience, University Hospital Düsseldorf, 40225 Düsseldorf, Germany; 4 Institute of Neuroscience and Medicine, Brain and Behaviour (INM-7), Research Centre Jülich, 52428 Jülich, Germany; 5 Helmholtz Institute for Biomedical Engineering, Stem Cell Biology and Cellular Engineering, RWTH Aachen University Medical School, 52074 Aachen, Germany

**Keywords:** Forensic age estimation, Epigenetic age estimation, DNA methylation, Children with growth disorders

## Abstract

Age estimation based on DNA methylation (DNAm) can be applied to children, adolescents and adults, but many CG dinucleotides (CpGs) exhibit different kinetics of age-associated DNAm across these age ranges. Furthermore, it is still unclear how growth disorders impact epigenetic age predictions, and this may be particularly relevant for a forensic application. In this study, we analyzed buccal mucosa samples from 95 healthy children and 104 children with different growth disorders. DNAm was analysed by pyrosequencing for 22 CpGs in the genes *PDE4C*, *ELOVL2*, *RPA2*, *EDARADD* and *DDO*. The relationship between DNAm and age in healthy children was tested by Spearman’s rank correlation. Differences in DNAm between the groups “healthy children” and the (sub-)groups of children with growth disorders were tested by ANCOVA. Models for age estimation were trained (1) based on the data from 11 CpGs with a close correlation between DNAm and age (*R* ≥ 0.75) and (2) on five CpGs that also did not present significant differences in DNAm between healthy and diseased children. Statistical analysis revealed significant differences between the healthy group and the group with growth disorders (11 CpGs), the subgroup with a short stature (12 CpGs) and the non-short stature subgroup (three CpGs). The results are in line with the assumption of an epigenetic regulation of height-influencing genes. Age predictors trained on 11 CpGs with high correlations between DNAm and age revealed higher mean absolute errors (MAEs) in the group of growth disorders (mean MAE 2.21 years versus MAE 1.79 in the healthy group) as well as in the short stature (sub-)groups; furthermore, there was a clear tendency for overestimation of ages in all growth disorder groups (mean age deviations: total growth disorder group 1.85 years, short stature group 1.99 years). Age estimates on samples from children with growth disorders were more precise when using a model containing only the five CpGs that did not present significant differences in DNAm between healthy and diseased children (mean age deviations: total growth disorder group 1.45 years, short stature group 1.66 years). The results suggest that CpGs in genes involved in processes relevant for growth and development should be avoided in age prediction models for children since they may be sensitive for alterations in the DNAm pattern in cases of growth disorders.

**Supplementary Information:**

The online version contains supplementary material available at 10.1007/s00414-022-02826-w.

## Introduction

Age estimation based on DNA methylation (DNAm) is about to become firmly established in the forensic methodical repertoire [1–3]. Current research questions particularly concern methodical aspects, the accuracy of age estimates and age-independent factors that may influence DNAm [4–6].

More and more studies focus on DNAm in children and adolescents. Many CpGs in genes that are comprised in models for forensic age estimation exhibit different kinetics of DNAm in children/adolescents, as compared to adults [7–10]. Most probably, this has to be attributed to molecular processes related to growth and development; an “accelerated” epigenetic aging goes along with faster physical development [11]. Despite these differences between non-adults and adults, the data published so far indicate that the relationship between DNAm and age may nevertheless be close within the group of healthy children and adolescents [7–10]. This comparably close relationship is surprising, considering that the complex processes of growth and development during childhood and adolescence are subdued to numerous influences, e.g. genetics, the individual constitutional disposition, endocrinologic factors, diet and diseases [12].

Furthermore, the question arises, if models for age estimation based on DNAm data from healthy children and adolescents can also be applied to young individuals with growth disorders.

“Growth disorders” is a generic term that encompasses many different syndromic and non-syndromic phenotypes. “Short stature” and “tall stature” are defined as height below the 2.5th or above the 97.5th percentile of a population [13]. It has been reported that more than 3200 genetic variants at more than 700 genomic loci influence growth and adult stature in subjects of European ancestry [14, 15]. These variants usually involve noncoding DNA intergenic or intronic sequences. The intergenic nucleotide variants often are the sites of epigenetic regulation of the expression of the associated genes [12, 14, 16]. Identified height-influencing genes are, for example, involved in cellular function and metabolic processes at the growth plate or are being assigned to the growth hormone axis; gene variants that affect stature in normal subjects include intracellular signalling, transcription, DNA repair, chromatin remodelling, collagen formation or paracrine signalling [14, 16, 17].

To what extent (epigenetic) alterations of one or more of these genomic loci affect growth and development remains subject of extensive research. For children with idiopathic short stature (ISS), a higher methylation status of the promoter region of the insulin like growth factor 1 gene has been detected and discussed as a contributor to the reduced body length [18]. DNAm profiling of children with short stature that were born small for gestational age revealed differentially methylated DNA regions that are said to be associated with imprinting disorders also affecting growth [19]. Studies on such developmental disorders that go along with alterations in body length demonstrate a crucial pathogenetic role for DNAm; in fact, typical methylation signatures have been identified that enable the diagnosis of these disorders on an epigenetic basis [20–22].

Therefore, it appeared even more relevant to test whether the age-dependent patterns of DNAm of genes that are used in models for forensic age estimation may be altered in children with growth disorders.

We investigated DNAm in buccal mucosa samples from 104 children and adolescents with growth disorders and compared the results with DNAm data from 95 healthy young individuals. DNAm analysis by pyrosequencing was performed for amplicons of the genes *PDE4C*, *ELOVL2*, *RPA2*, *EDARADD* and *DDO.* Furthermore, an epigenetic age-prediction model was trained for 11 age-associated CpGs for healthy children and adolescents, which was subsequently applied to the young individuals with growth disorders.

## Material and methods

### Analysed samples

A detailed list of the study participants can be found in the supplementary material. Children/adolescents were only included after written informed consent of their legal guardians.

Buccal mucosa samples were collected from 95 healthy children/adolescents (healthy group: ages between 0.42 and 18 years; 51 females, 44 males) and 104 children/adolescents that were patients of the paediatric clinic of the University Hospital Düsseldorf, suffering from a growth disorder (growth disorder group: ages between 1 and 17 years; 48 females, 56 males). The only condition for inclusion into the study was the diagnosis “growth disorder”, independent of the specific clinical diagnosis/aetiology; however, the collective comprised predominantly cases with short stature.

In detail, the growth disorder group is composed of the following subgroups:

- 76 children/adolescents with short stature (short stature group, body length under the 2.5th percentile: ages between 1 and 15 years; 28 females, 48 males)
  - 33 children/adolescents with idiopathic short stature (idiopathic short stature group: ages between 2 and 15 years; 11 females, 22 males)
  - 29 children/adolescents with short stature of endocrinologic aetiology (endocrinologic group: ages between 3 and 15 years; 8 females, 21 males)
  - 14 children/adolescents with short stature of genetic aetiology (genetic group: ages between 1 and 11 years; 9 females, 5 males)
- 28 children/adolescents without short stature (body length between 2.5th and 97.5th percentiles or over 97.5th percentile), comprising clinical diagnoses like precocious puberty, tall stature, failure to thrive (non-short stature group: ages between 1 and 17 years; 20 females, 8 males).

### DNA extraction, quantification and bisulphite conversion

Genomic DNA was extracted from buccal swab samples of both groups using the NucleoSpin® Tissue Kit from Macherey–Nagel (North Rhine-Westfalia/Germany) according to the manufacturer’s protocol. Lysis was performed overnight at 56 °C. Elution of DNA was performed in 100 µl BE buffer (part of the extraction kit). Quantification was performed according to the manufacturer’s protocol using either the Applied Biosystems™ 7500 Real-Time PCR System and Quantiplex® Pro Kit (Qiagen, North Rhine-Westfalia/Germany) or the QuantiFluor dsDNA Sample Kit (Promega, Wisconsin/USA) and Quantus Fluorometer (Promega, Wisconsin/USA).

For bisulphite conversion of DNA, either the EZ DNA Methylation-Gold™ Kit (Zymo Research, California/USA) or the EpiTect Fast DNA Bisulphite Kit (Qiagen, North Rhine-Westfalia/Germany) was used according to the manufacturer’s protocol. Positive controls that were included in each analysis did not reveal relevant differences with regard to the utilized kit. Since buccal swabs from children often contain only little amounts of DNA, the analysis parameters were adapted according to the kits’ manuals. If possible, the optimal amount of 200 to 500 ng of input DNA was used; in some cases, lower amounts had to be accepted, but in no case less than 10 ng per reaction volume was used, which is a critical threshold [23].

### DNA methylation analysis by pyrosequencing (22 CpGs located in the genes PDE4C, RPA2, ELOVL2, DDO and EDARADD)

For amplification, CpG-specific PCRs were performed using either the HotStar-Taq kit (Qiagen, North Rhine-Westfalia/Germany) or the PyroMark PCR kit (Qiagen, North Rhine-Westfalia/Germany) according to the manufacturer’s protocol. Primer sequences for 22 CpG sites that were already successfully used in a preceding project [24] and that are reported to show an age-correlated DNAm status in cells obtained by buccal swabs were taken from the original papers [25–27]. Information about the primer sequences, the chromosome location of the CpG sites and the PCR conditions can be found in the supplementary material. For subsequent pyrosequencing, 10–20 µl of the biotinylated PCR product was immobilized on 1 μl streptavidin Sepharose™HP beads (GE Healthcare, Illinois/USA). Sequencing primers were designed as previously described [25–27]. Pyrosequencing was performed using the PyroMark Q24 Advanced CpG Reagents Kit (Qiagen, North Rhine-Westfalia/Germany) and the PyroMark Q24 Advanced System (Qiagen, North Rhine-Westfalia/Germany). Positive and negative controls were also included and evaluated after each run, and agarose gels were checked for the presence and the right length of PCR products. Pyrosequencing was considered reliable when sequencing was correct (e.g. peaks not too small or missing), and pyrograms reached a pre-defined threshold.

Twenty-six samples of the healthy group (and 109 samples of a previous study) were analysed at least twice. The difference between the obtained DNAm did not exceed 3% as long as the pyrograms did not contain error messages. With regard to costs and practicability, the remaining samples were therefore analysed only once.

### Relationship between DNAm and age for 22 CpG sites in healthy children and adolescents

The relationship between DNAm and age in the healthy group was tested by Spearman’s rank correlation. For all CpGs (located in the genes *PDE4C*, *ELOVL2*, *RPA2*, *EDARADD* and *DDO*), Spearman’s correlation coefficients (*R*) were calculated. CpGs revealing a close correlation between DNAm and age (*R* > 0.75) were used for the development of age prediction models (so called “11 CpGs models”, see below).

### Testing for differences in DNAm between individuals with and without growth disorders

DNAm differences between the investigated groups of children/adolescents were tested by ANCOVA; at a *p*-value < 0.05, results were considered significant. In detail, the growth disorder group (*n* = 104), the short stature group (*n* = 76) and the non-short stature group (*n* = 28) were each tested versus the healthy group (*n* = 95). We also tested for differences between male (*n* = 54) and female (*n* = 48) individuals within the growth disorder group.

Though it is well known that BMI can influence DNAm [28], we refrained from testing for differences between children/adolescents with under-, over- or normal weight, since these data were only available for the growth disorder group resulting in numbers too small for an adequate calculation (underweight *n* = 17, overweight *n* = 14, normal weight *n* = 73; see also supplementary material).

### Age estimation, model validation and group comparison

The key challenge for the current investigation was to reconcile training of the predictive models in the available cohort of healthy children while at the same time comparing their performance between (also) healthy children and those with growth disorders. In this context, it is important to stress that testing the trained models in the same subjects that were used for training is invalid, as model training and evaluation need to be strictly separated. In addition, we note that the age and sex distribution of the target groups (growth disorder and short stature) deviated from the available controls, representing a potential confound for the interpretation of any difference in predicted age.

To overcome these challenges, we used a matched subsampling approach with Matlab® 2021 and the “Statistics and Machine-Learning Toolbox”. In particular, we drew 25,000 (unique) paired subsamples from the reference cohort and one target cohort, respectively, which were well matched to each other in terms of age and sex (both *p* > 0.3). Per cohort, the number of individuals drawn for these subsamples represented 10% (rounded upwards) of the smaller of the two sets, i.e. reference and target. The selected subsamples were then set aside as the test data so that the subjects drawn from the reference cohort were not used for training but remained unseen to the algorithm in the same way as the (matched) subsample from the target cohort. That is, we drew from each cohort the same number of subjects in such a way that the two selected subsamples were closely matched for age and gender while ensuring that at least 90% of the reference cohort was available for training.

Repeating this setup 25,000 times then allows deriving distributions of prediction accuracy (how well did models trained on the remaining reference cohort generalize to the unseen reference subjects and those with a growth disorder) and bias (systematic difference in predicted age). The former was evaluated by the mean absolute errors (MAEs). The results for the healthy children test samples were then compared against those for the growth disorder test samples that were matched regarding the number of samples, age and sex. The latter, biases resulting in a systematic over- or underestimation of the growth disorder test samples, was quantified by the mean age gap (i.e. the difference between the estimated and the chronological age) and again compared across groups.

**Fig. 1:**
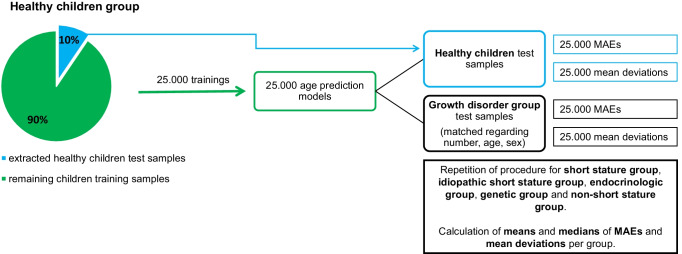
Illustration of age predictor modelling and performance procedure. Training of 25.000 models and performance testing on extracted healthy children test samples as well as on matched test samples from diseased children was performed in the same way for all growth disorder (sub-)groups. MAE mean absolute error

This approach was applied to all growth disorder (sub-)groups. For a graphic illustration of modelling and performance procedures, see Fig. 1.

The age prediction models themselves were trained on the part of the healthy reference cohort that was not set aside for testing, using a random forest algorithm using (chronological) age as the continuous target variable as well as the CpG information and sex as features for prediction. In a first step, we trained 11 CpGs models; in a second step, “robust” models were trained by using only the robust 5 CpG sites that did not present significant differences between healthy children and children with growth disorders (so called “5 CpGs models”). The prediction forest consisted of 1000 individual trees that were built from bootstrap-samples of the training dataset using the curvature test. This test selected the split predictor that minimizes the *p*-value of chi-square tests of independence between each predictor, i.e. feature, and the response, i.e. age.

The detailed set parameters were as follows:

mdl = TreeBagger(trees,data(testFold(i,:) =  = 0,:),Target(testFold(i,:) =  = 0),'CategoricalPredictors',1,'Method','regression','Surrogate','all','MinLeafSize',1,…

'PredictorSelection','curvature','options',statset('useparallel',true),'OOBPrediction','on');

Minimum Leaf Size 1.

Split selection: Curvature test—selects the split predictor that minimizes the *p*-value of chi-square tests of independence between each predictor and the response.

With regard to single missing values: surrogate splits—when the value of the optimal split predictor for an observation is missing; if you specify to use surrogate splits, the software sends the observation to the left or right child node using the best surrogate predictor.

## Results

### Close correlations between DNAm and age at 11 of 22 CpG sites in the healthy group

The analysis of DNAm levels at the 22 CpG sites in the healthy group resulted in correlation coefficients (Spearman R) between − 0.44 (*DDO*) and 0.87 (*ELOVL*2/CpG3 and 6) (Table 1). For half of the 22 CpG sites, R was lower than 0.75; these sites were excluded from further analysis, i.e. age estimation modelling. The remaining 11 CpGs with a close correlation between DNAm and age were included in the11 CpGs models. Figures depicting analysis results versus donors’ age sorted by (sub-)groups from these 11 CpGs are provided in the supplementary material.

**Table 1:**
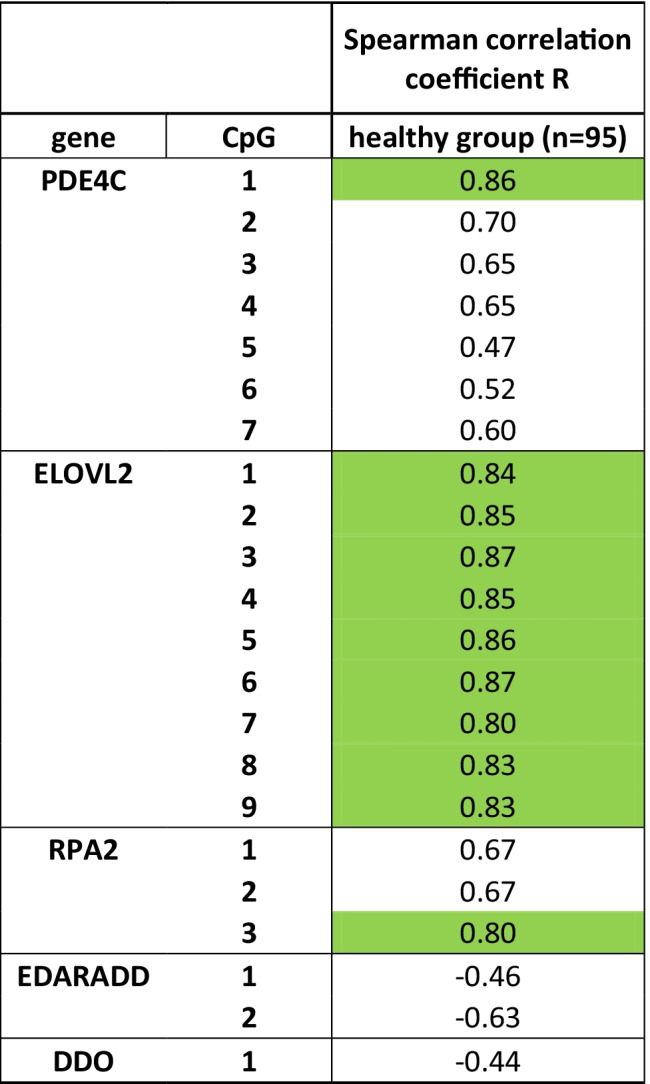
Analysed CpGs (in the genes *PDE4C*, *ELOVL2*, *RPA2*, *EDARADD* and *DDO*) and Spearman’s correlation coefficients (*R*) for the relationship between DNA methylation and age in healthy children. CpGs with *R* > 0.75 were included in age prediction models and are marked in green

### Significant differences in DNAm between individuals with and without growth disorders

ANCOVA revealed significant differences (with *α* < 0.05) at 11 CpG sites in all genes between the healthy group and the total growth disorder group, and at 12 CpG sites in all genes between the healthy group and the short stature subgroup; in the non-short stature group, only 3 CpG sites in the genes *PDE4C*, *ELOVL2* and *RPA2* presented significant differences. The lowest *p*-values were found for *RPA2*/CpG3, *ELOVL2*/CpG7 and *ELOVL2*/CpG3 (Table 2). No significant differences were found when testing male vs female individuals within the growth disorder group.

**Table 2:**
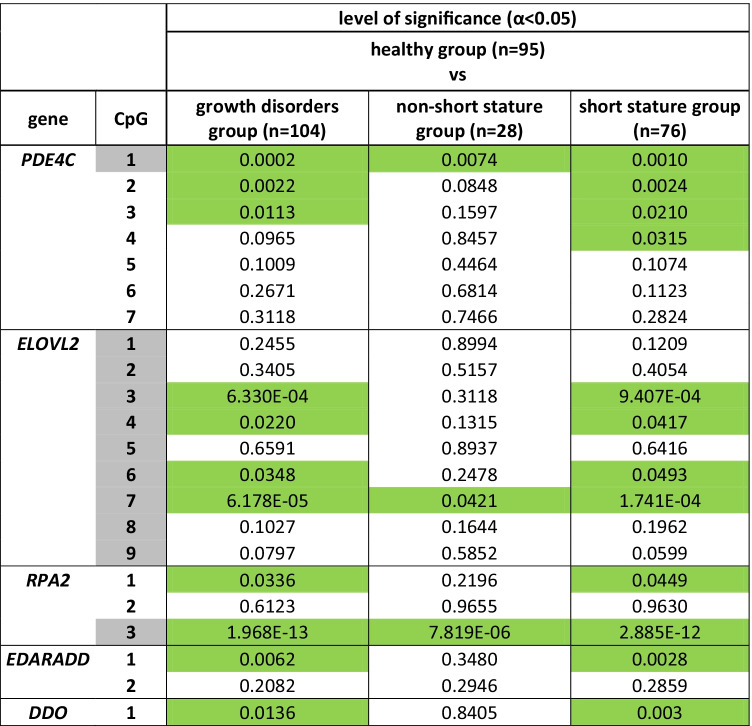
Differences in DNAm between healthy group and growth disorder group as well as short stature group as revealed by ANCOVA. Significant differences (*α* < 0.05) are marked in green. CpG sites that were chosen for age prediction models are marked in light grey

Six of the 11 CpG sites that showed significant differences in DNAm when comparing the healthy group with the growth disorder (sub-)groups are among those with a close correlation between DNAm and age (*R* > 0.75); the remaining 5 sites with a close correlation between DNAm and age (CpG1, CpG2, CpG5, CpG8 and CpG9 in *ELOVL2*) appear to be robust by exhibiting no significant differences between the two groups. These five CpGsites were comprised in the 5 CpGs models.

### Age prediction by models based on healthy children training data (“11 CpGs models”) reveal a tendency for overestimating the ages of children with growth disorders, as compared to age- and sex-matched healthy children test samples

**Fig. 2:**
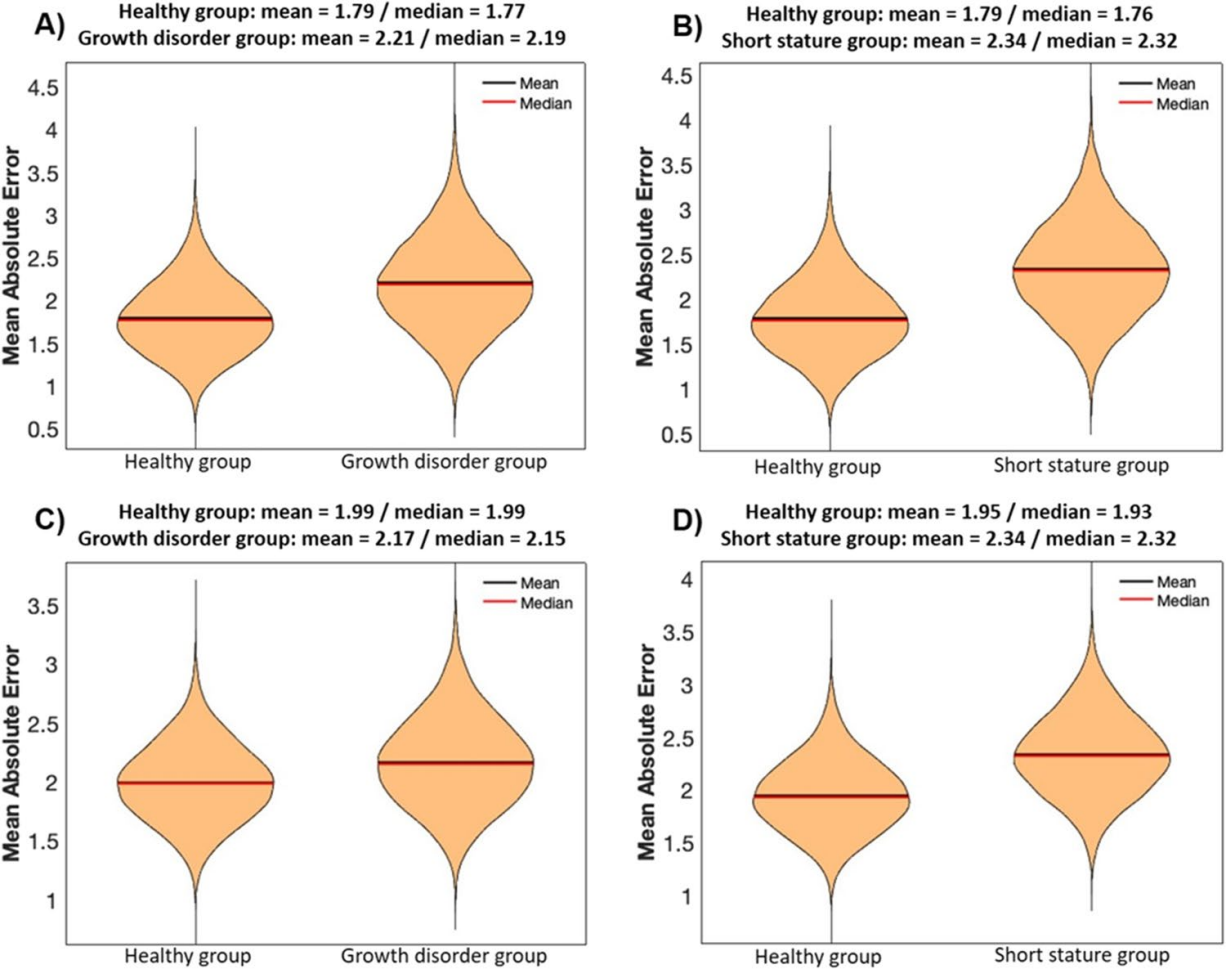
Mean absolute errors (MAE, in years) of 25,000 age estimations based on healthy children training samples (90% of the total healthy group sample number each) and performed on healthy children test samples (healthy group: extracted 10% of the total healthy group samples) as well as on test samples of the growth disorder group and the short stature group (each matched regarding sample number, age and sex). **A**, **B** 11 CpGs models. **C**, **D** 5 CpGs models

The means and medians of the MAEs of age estimation based on the 11 CpGs models calculated in 25,000 runs for the test samples of the growth disorder group (mean 2.21 years, median 2.19 years) were slightly higher than those for the age- and sex-matched healthy children test samples (mean 1.79 years, median 1.77 years). With regard to the 25,000 runs for test samples of the short stature group (mean 2.34 years, median 2.32 years), the differences were even slightly higher when compared to age- and sex-matched healthy children test samples (mean 1.79 years, median 1.76 years). Means and medians of the MAEs of the 5 CpGs models that were calculated in the same way showed comparable values (Fig. 2). The same accounts for the results of the subgroups idiopathic short stature, genetic aetiology, endocrinologic aetiology and non-short stature: the calculated means and medians did not show obvious differences (Table 3).

**Table 3:** Performance of “11 CpGs” and “5 CpGs” age prediction models, trained on healthy children training data: overview of means and medians of mean absolute errors (MAEs) and age deviations for all growth disorder (sub-)groups. Test samples = test samples of the respective growth disorder (sub-)group (results presented in bold numbers); Reference samples healthy group = pre-extracted test samples of the healthy children group

Group	MAE (years) 11 CpGs models		MAE (years) 5 CpGs models		Age deviation (years) 11 CpGs models		Age deviation (years) 5 CpGs models	
	Mean	Median	Mean	Median	Mean	Median	Mean	Median
Growth disorder group (*n* = 104)								
Test samples	**2.21**	**2.19**	**2.17**	**2.15**	**1.85**	**1.84**	**1.45**	**1.45**
Reference samples healthy group	1.79	1.77	1.99	1.99	0.28	0.27	0.21	0.20
Short stature group (*n* = 76)								
Test samples	**2.34**	**2.32**	**2.34**	**2.32**	**1.99**	**1.99**	**1.66**	**1.66**
Reference samples healthy group	1.79	1.76	1.95	1.93	0.52	0.51	0.68	0.68
Idiopathic short stature group (*n* = 33)								
Test samples	**2.52**	**2.49**	**2.47**	**2.48**	**2.15**	**2.14**	**1.77**	**1.77**
Reference samples healthy group	1.81	1.78	1.99	1.98	0.62	0.61	0.68	0.67
Endocrinologic group (*n* = 29)								
Test samples	**2.37**	**2.36**	**2.36**	**2.36**	**1.98**	**1.99**	**1.52**	**1.52**
Reference samples healthy group	1.92	1.90	2.04	2.03	0.58	0.58	0.62	0.62
Genetic group (*n* = 14)								
Test samples	**2.28**	**2.29**	**2.21**	**2.20**	**1.99**	**2.00**	**1.82**	**1.82**
Reference samples healthy group	1.85	1.82	2.10	2.08	0.88	0.88	1.05	1.05
Non-short stature group (*n* = 28)								
Test samples	**2.06**	**2.05**	**2.10**	**2.10**	**1.76**	**1.76**	**1.45**	**1.45**
Reference samples healthy group	1.76	1.73	1.97	1.96	0.11	0.10	0.15	0.14

**Fig. 3:**
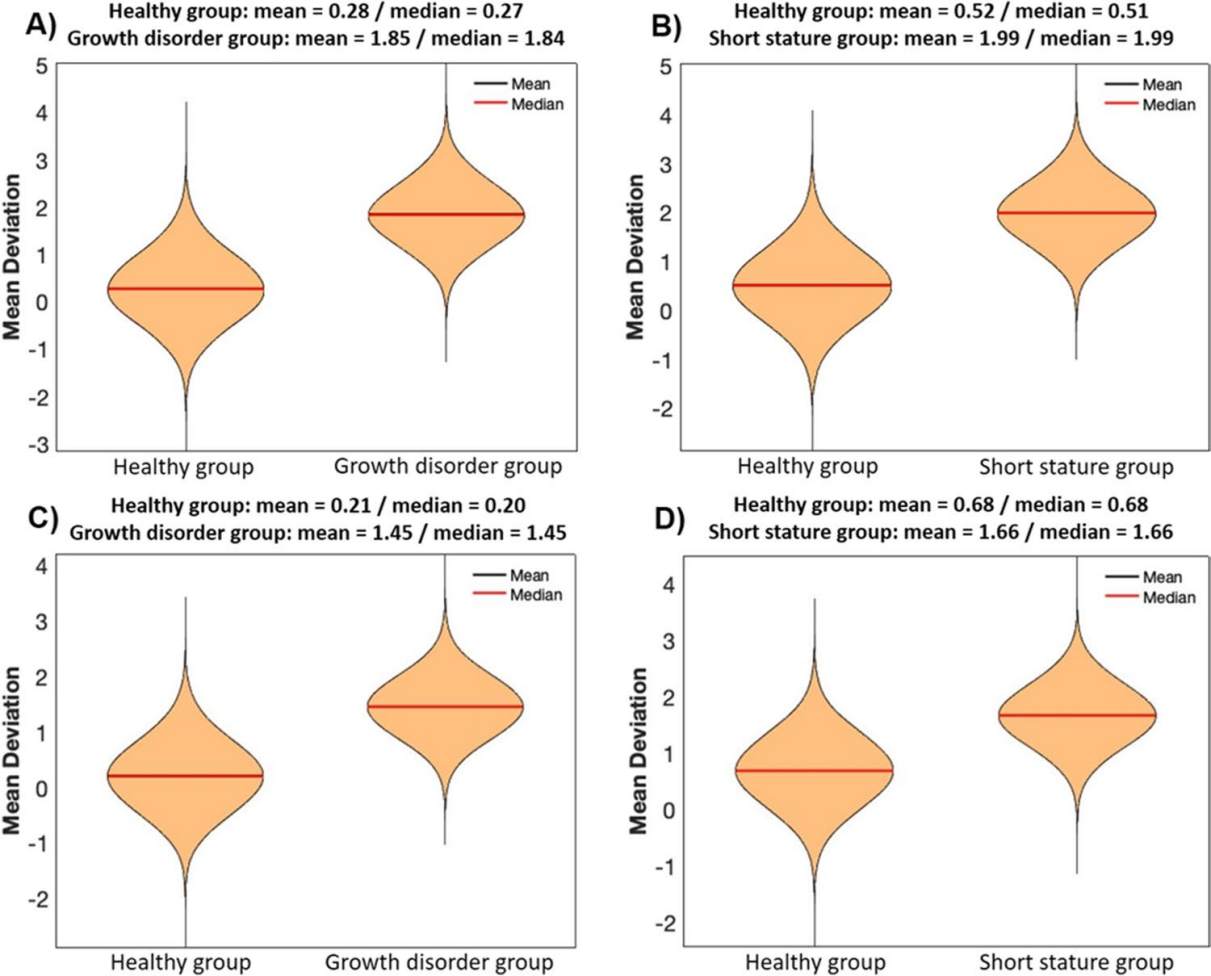
Mean deviation of the age gaps (difference between estimated and chronological ages in years) of 25,000 age estimations based on healthy children training samples (90% of the total healthy group sample number each) and performed on healthy children test samples (healthy group: extracted 10% of the total healthy group samples) as well as on test samples of the growth disorder group and the short stature group (each matched regarding sample number, age and sex). **A**, **B** 11 CpGs models. **C**, **D** 5 CpGs models

The mean deviations of the age gaps (the differences between estimated and chronological ages) revealed a clear tendency for overestimation of ages in the growth disorder group (mean 1.85 years, median 1.84 years; healthy group mean 0.28 years and median 0.27 years) as well as in the short stature group (mean 1.99, median 1.99 years; healthy group mean 0.52 years and median 0.51 years) in the 11 CpGs models. In the 5 CpG models, on the other hand, the age gaps were clearly smaller for the growth disorder group (mean 1.45 years, median 1.45 years; healthy group mean 0.21 years, median 0.20 years) and the short stature group (mean 1.66, median 1.66; healthy group mean 0.68, median 0.68) (Fig. 3). The same accounts for the growth disorder subgroups (Table 3).

## Discussion

### Methodical aspects

Since DNA samples of young individuals with growth disorders are difficult to obtain, we had to work with a rather small (*n* = 104 in the growth disorder group) and heterogeneous sample collection.

To tackle this challenge, we calculated numerous models when testing the performance of age prediction on the healthy group and the growth disorder group, as well as its subgroups. Twenty-five thousand models were calculated which were then used for age prediction on matching test samples of the total healthy group and one of the 6 growth disorder (sub-)groups. The resulting mean and median MAEs of the prediction runs provide a reliable impression of how well DNAm-based age predictors that have been trained on data derived from healthy children/adolescents work on test samples of individuals with growth disorders.

The study at hand was conducted with the primary aim to test the influence of growth disorders on DNAm and, in a forensic context, on DNAm-based age estimation in children and adolescents. We did not intend to establish a new final model for forensic age estimation. The calculated models only serve the purpose to test and illustrate the alleged influence.

### Biological aspects

Eleven of the 22 analysed CpGs in the genes *PDE4C*, *ELOVL2* and *RPA2* exhibited significant differences between young, healthy individuals and age- and sex-matched individuals with growth disorders. A final biological interpretation of the significant differences in 7 of these 11 CpGs when comparing individuals with and without growth disorders is, of course, not possible solely based on the presented data. However, it can be stated that the findings are in line with publications that focus on (epi)genetic factors in the pathophysiology of growth disorders.

It has been proposed that genes involved in fundamental cellular processes like intracellular signalling, transcription, DNA repair or chromatin remodelling play an important role in growth and in the pathophysiology of growth disorders [14, 29]. Against this background, it might not be too surprising that the analysed CpGs in the genes *PDE4C* and *ELOVL2* show a different methylation level in young individuals with growth disorders when compared to healthy children/adolescents, since both genes take part in such processes: PDE4C is a member of the phosphodiesterase superfamily and insofar involved in the control of intracellular levels of cyclic nucleotides, underlying a vast number of physiologic processes (e.g. [30]); ELOVL2 belongs to a group of enzymes that are vital for the elongation of fatty acids resulting in very long–chain fatty acids, major building blocks for various molecules (e.g. [31]). The finding of differences in DNAm patterns of the *RPA2* gene is even more interesting: RPA is a multifunctional protein that plays a role in DNA replication, repair, recombination and beyond [32], being essential for cell cycle progression. Upon DNA damage, RPA gains negative charges through phosphorylation at the N-terminus of RPA2 that carries the phosphorylation motif. If this gene would be regulated epigenetically and DNAm would play a role, it would be plausible that altered DNAm patterns may result in disorders of growth. But of course, this is merely a highly speculative hypothesis which is based only on the analytical results of single CpGs and which cannot be further substantiated.

### Forensic aspects

Our study proofs age-related DNAm at certain CpG sites in young individuals, meaning that DNAm-based age estimation can also be applied for children and adolescents and thus confirms results of other studies [7, 8].

However, our 11 CpGs models included 6 CpGs with significant differences in DNAm between healthy children and children with growth disorders. These differences were less obvious in the MAEs but presented themselves as a systemic upward “shift” in the age gaps. As a consequence, there was a clear tendency to overestimate the ages of children with growth disorders, with mean deviations of about + 1.85 years. That means, the inclusion of (too many of) such “sensible” CpGs in age predictors may lead to systematic errors in age estimation. Taking into account that, for example, about 3% of children in Germany meet the diagnostic requirements of short stature [33], these findings cannot be neglected in forensic practice. Optimized models for age estimation should aim at the inclusion of as many robust CpGs as possible; for growth disorders, such robust CpGs are for example CpG1, CpG2, CpG5, CpG8 and CpG9 in *ELOVL2*. Our 5 CpGs models which contained only these robust CpGs obviously performed somewhat better on test samples of diseased children (Fig. 3; Table 3).

In this respect, CpGs in genes involved in processes relevant for growth and development are more likely to be unsuitable for age prediction models since they seem to be especially sensitive for alterations in the DNAm pattern in cases of growth/developmental disorders.

## Supplementary Information

Below is the link to the electronic supplementary material.

Supplementary file1 (DOCX 925 kb)

Supplementary file2 (XLSX 100 kb)

Supplementary file3 (XLSX 11 kb)
