# Supplementary material for: Altered DNA methylation at age-associated CpG sites in children with growth disorders: impact on age estimation?
Source: Int J Legal Med. 2022 May 12;136(4):987–96. doi: 10.1007/s00414-022-02826-w (PMC9170667; doi:10.1007/s00414-022-02826-w)
Supplement: Supplementary file 1 — Supplementary file1 (DOCX 925 kb) [file 414_2022_2826_MOESM1_ESM.docx]

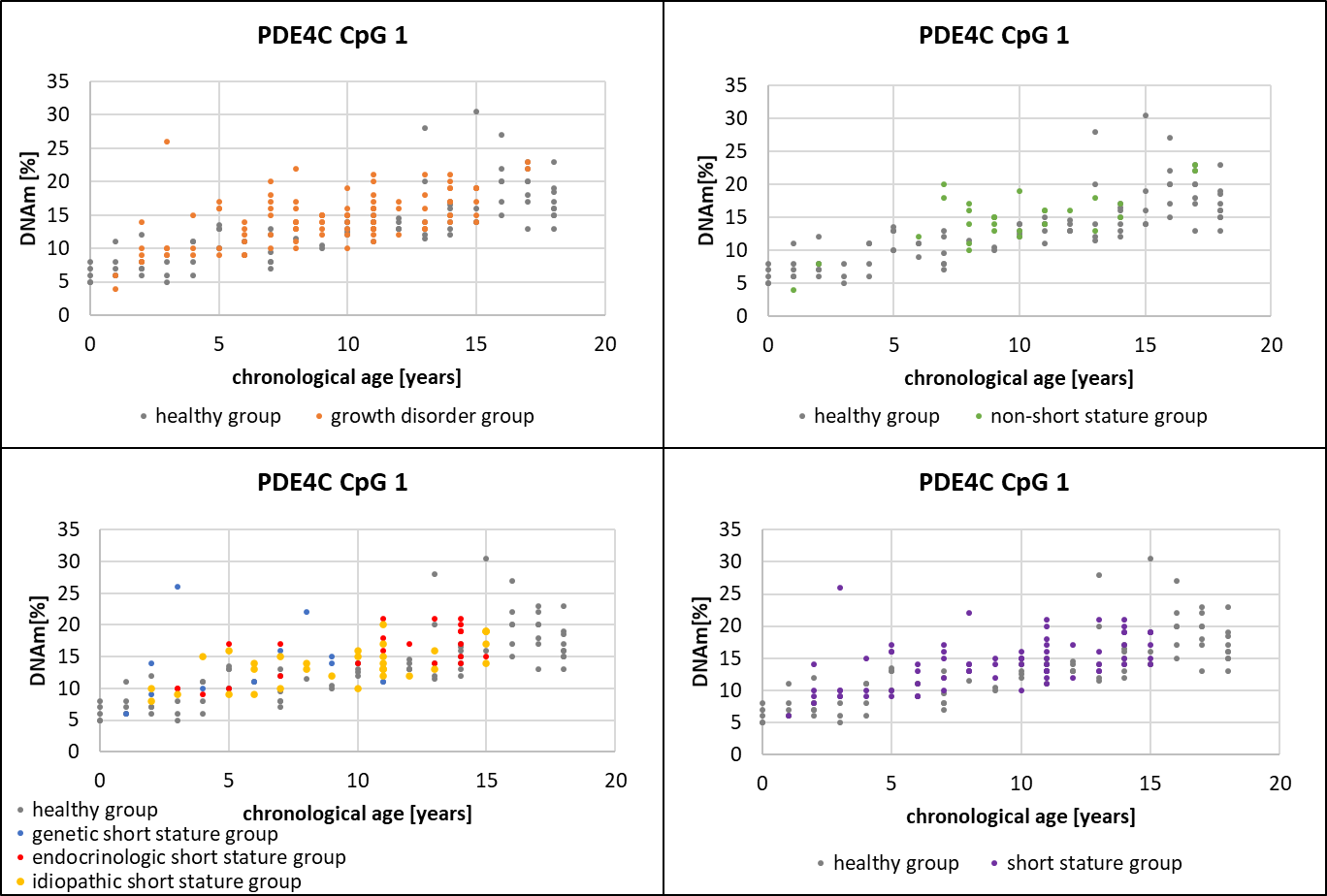


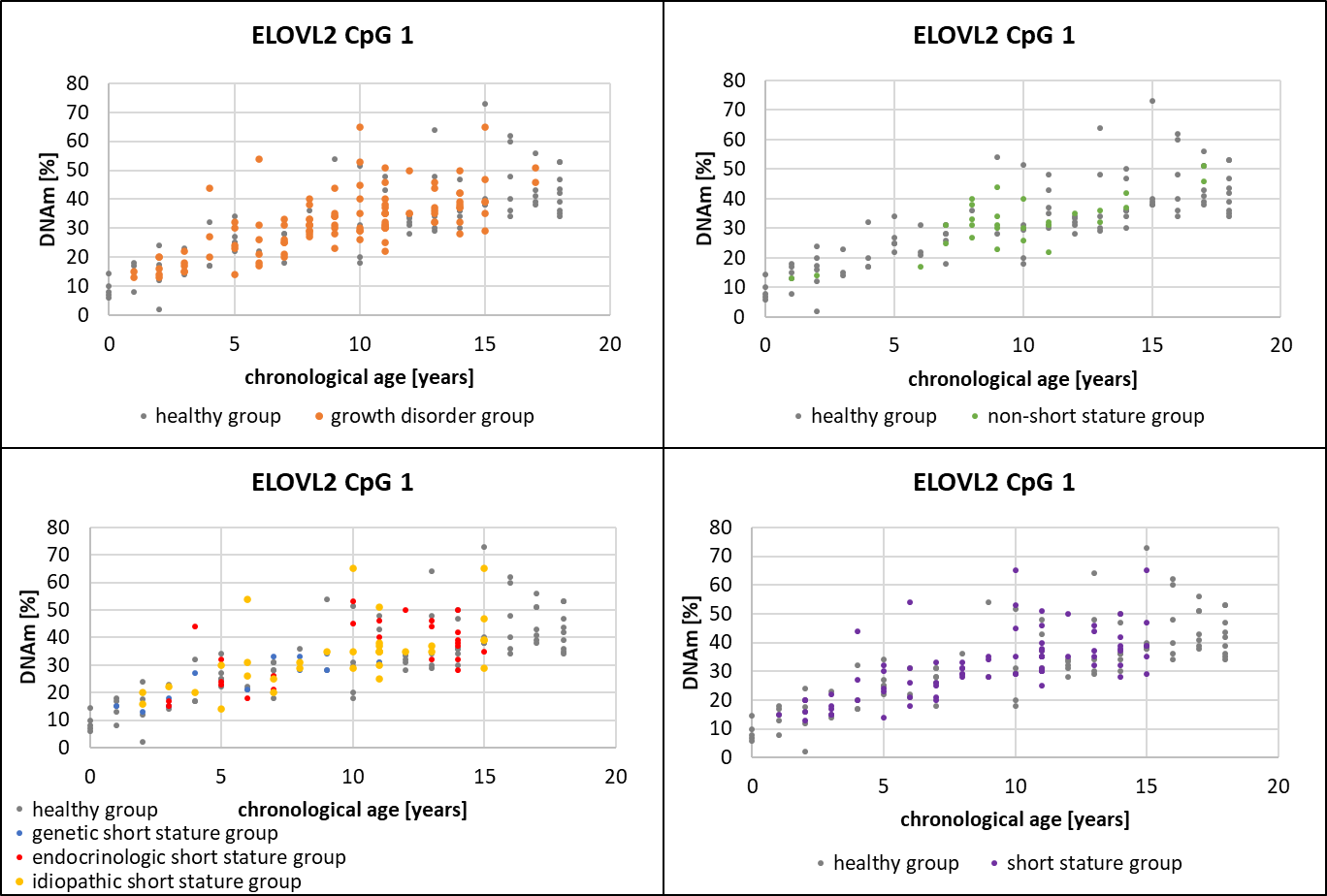


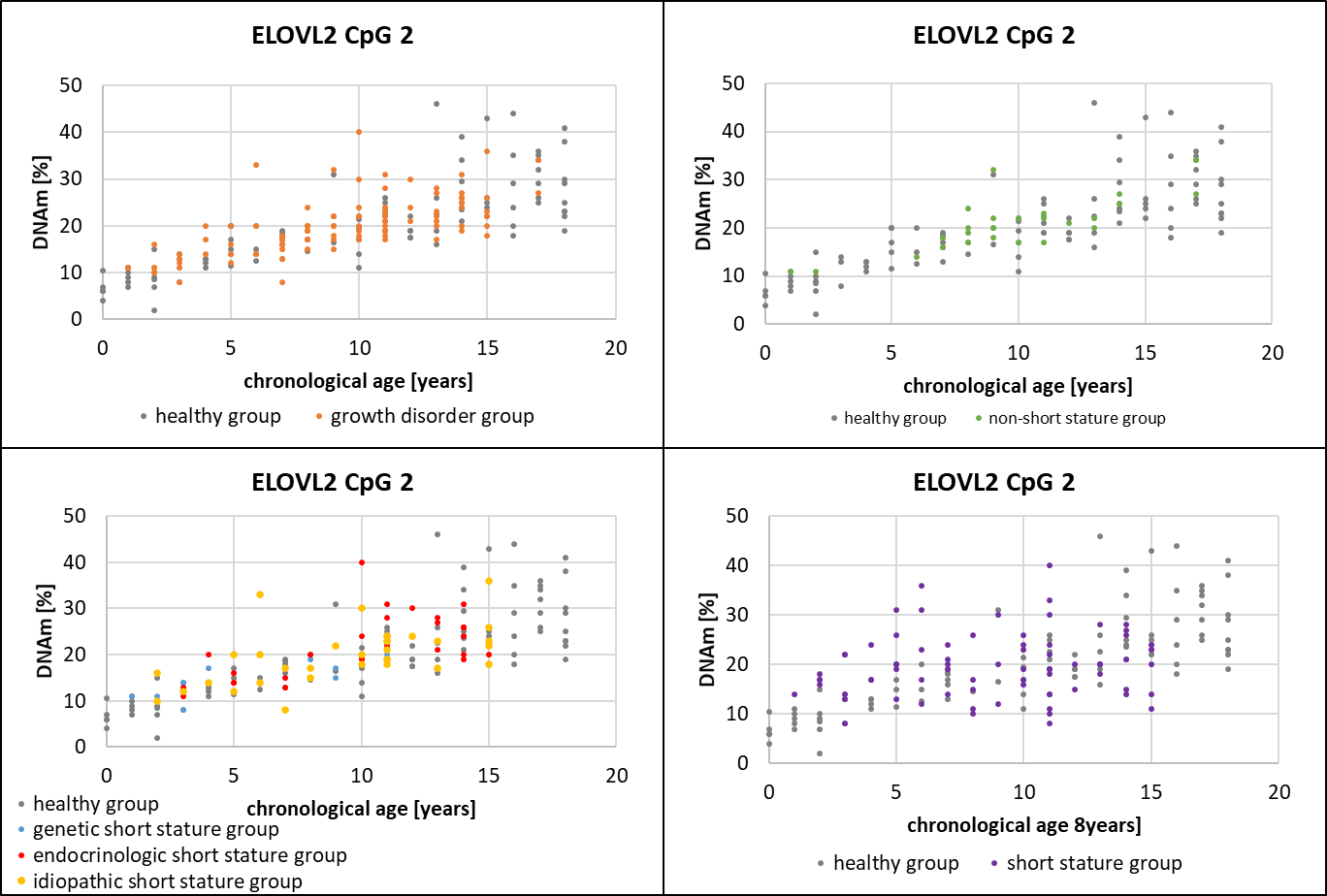


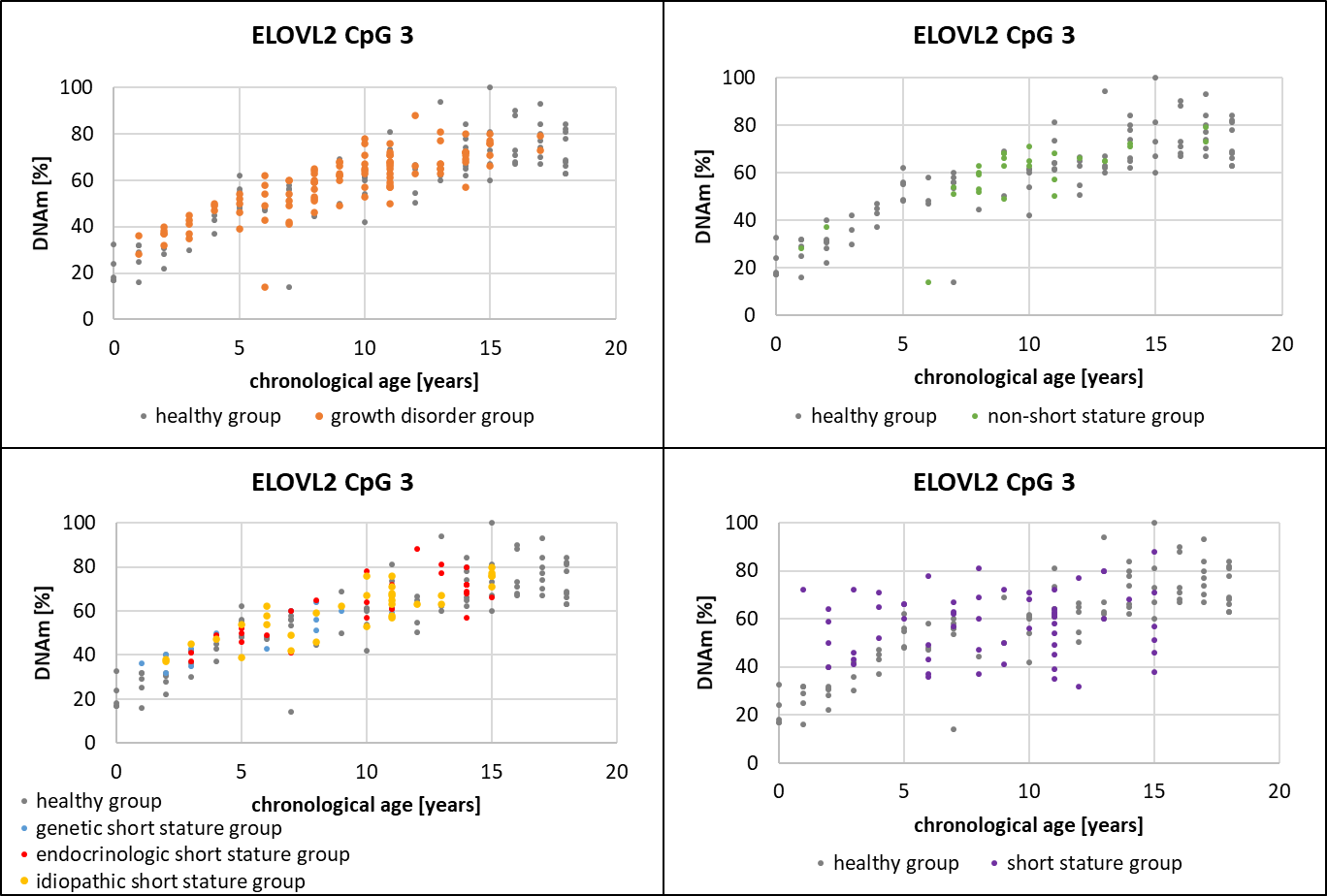


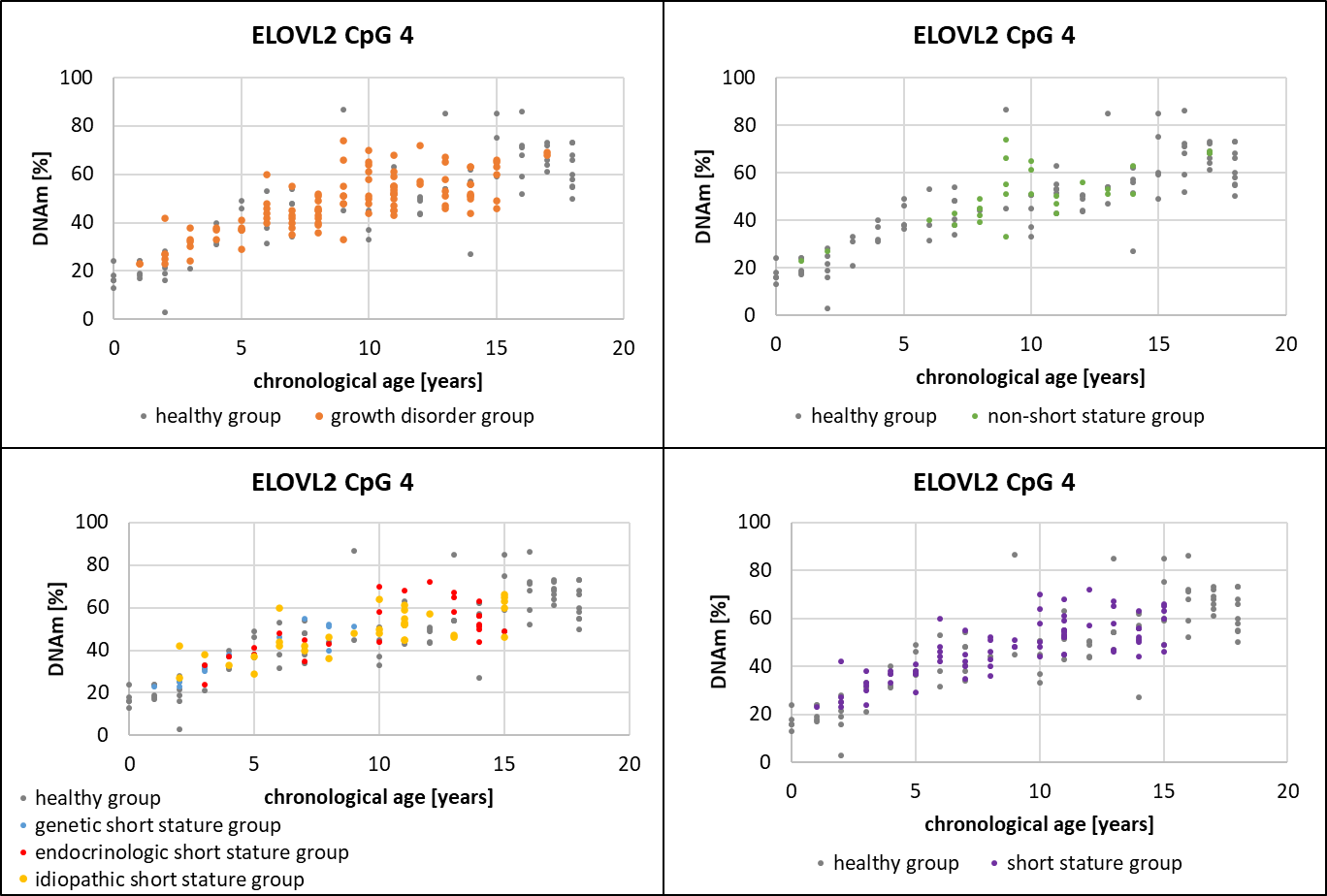


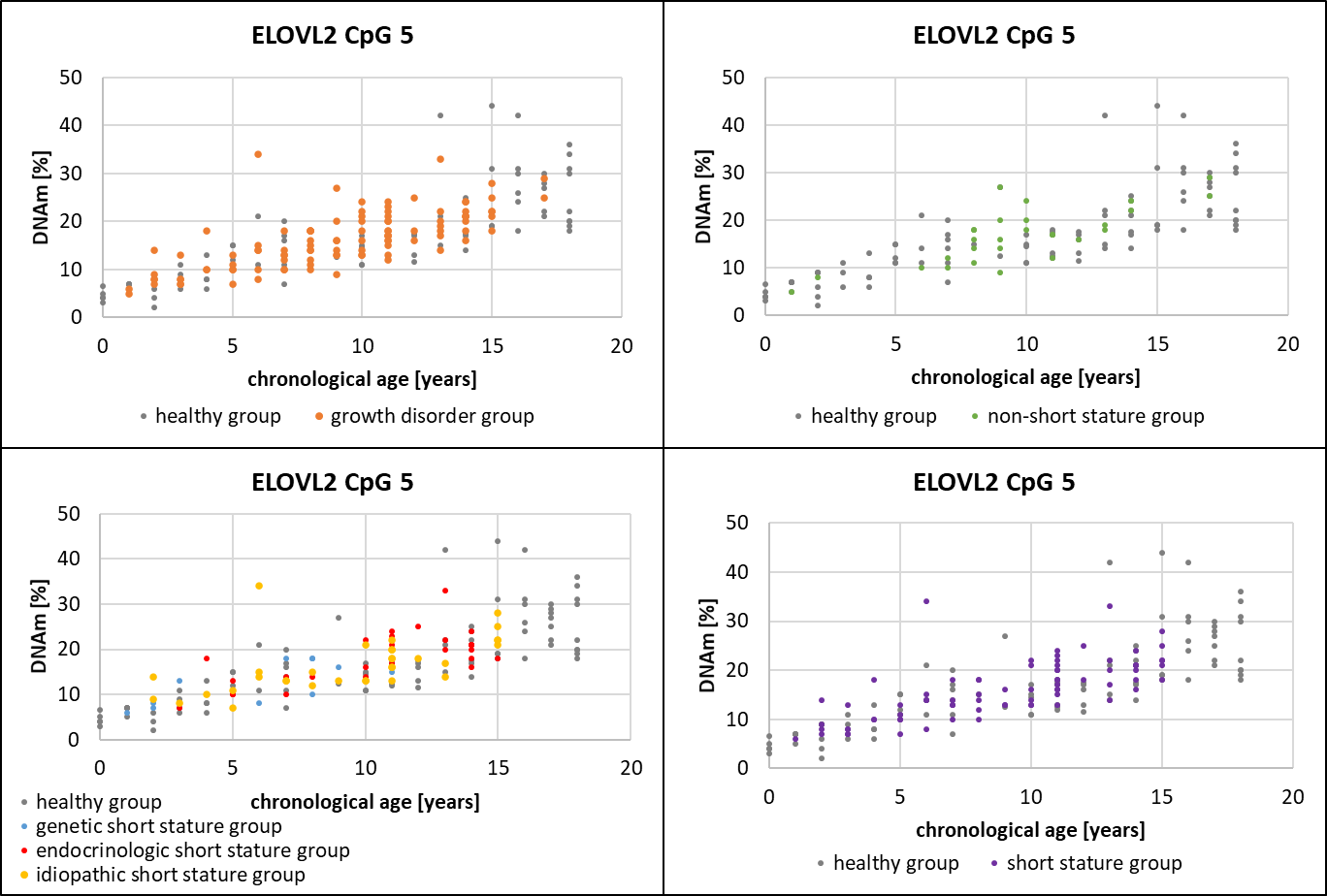


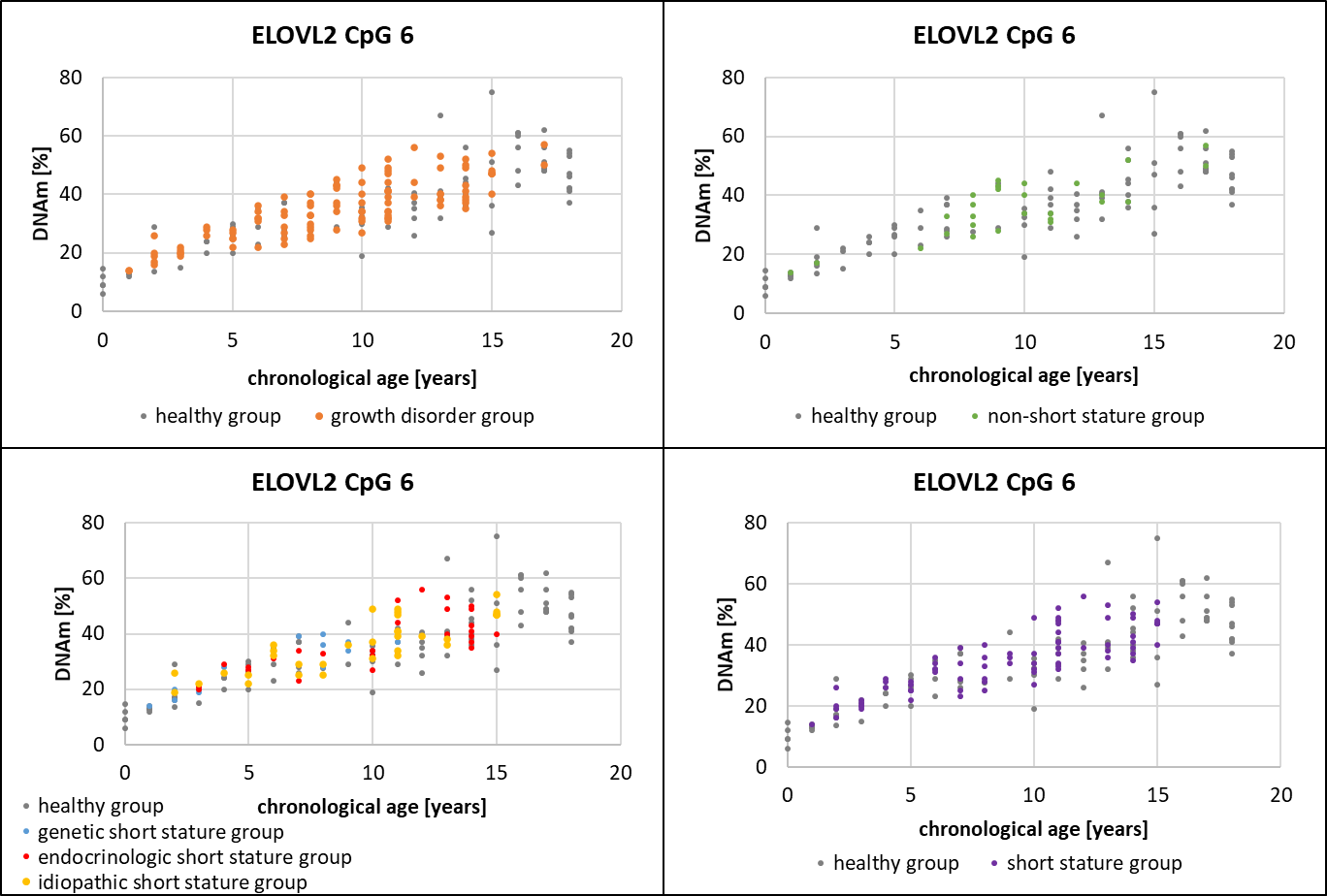


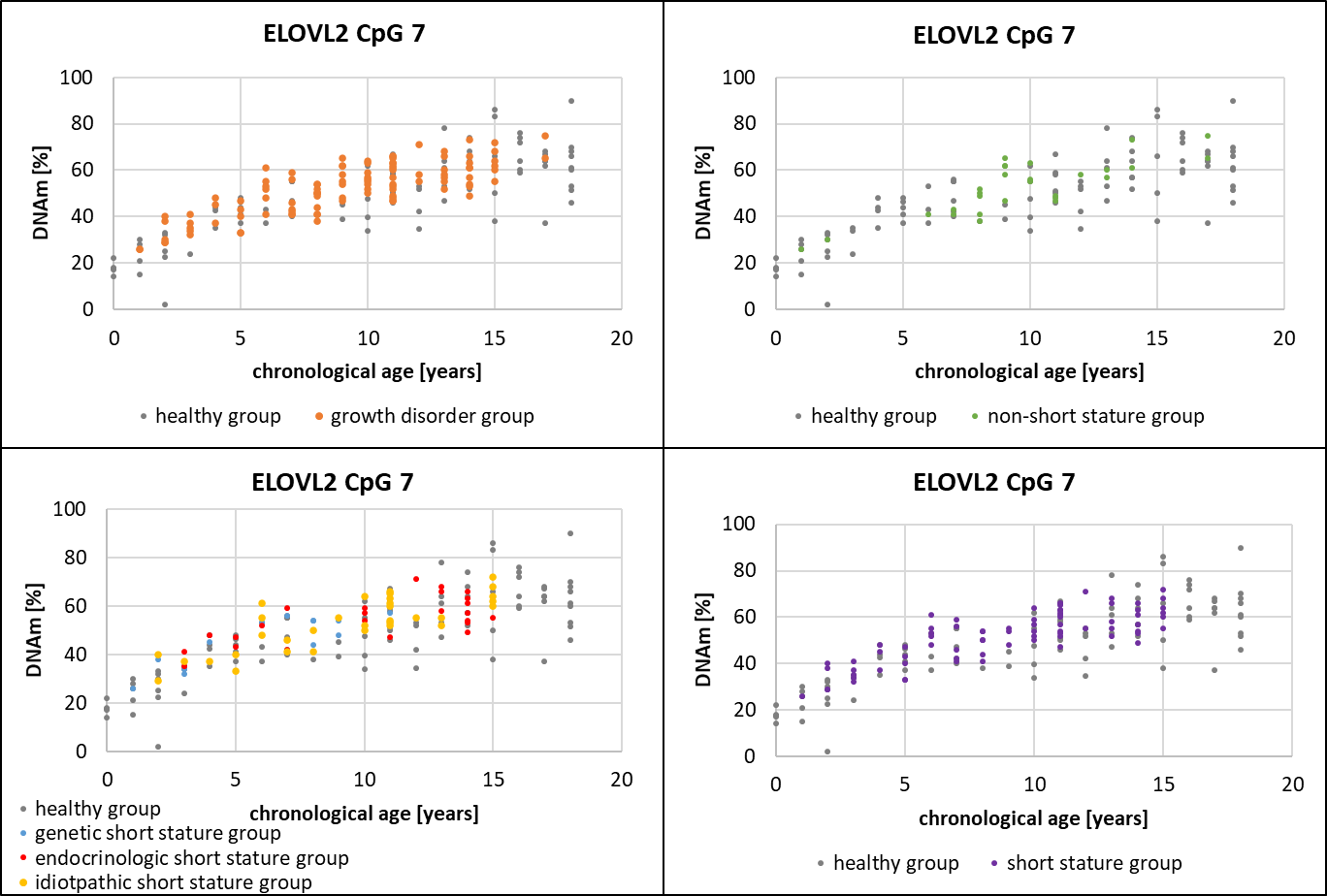


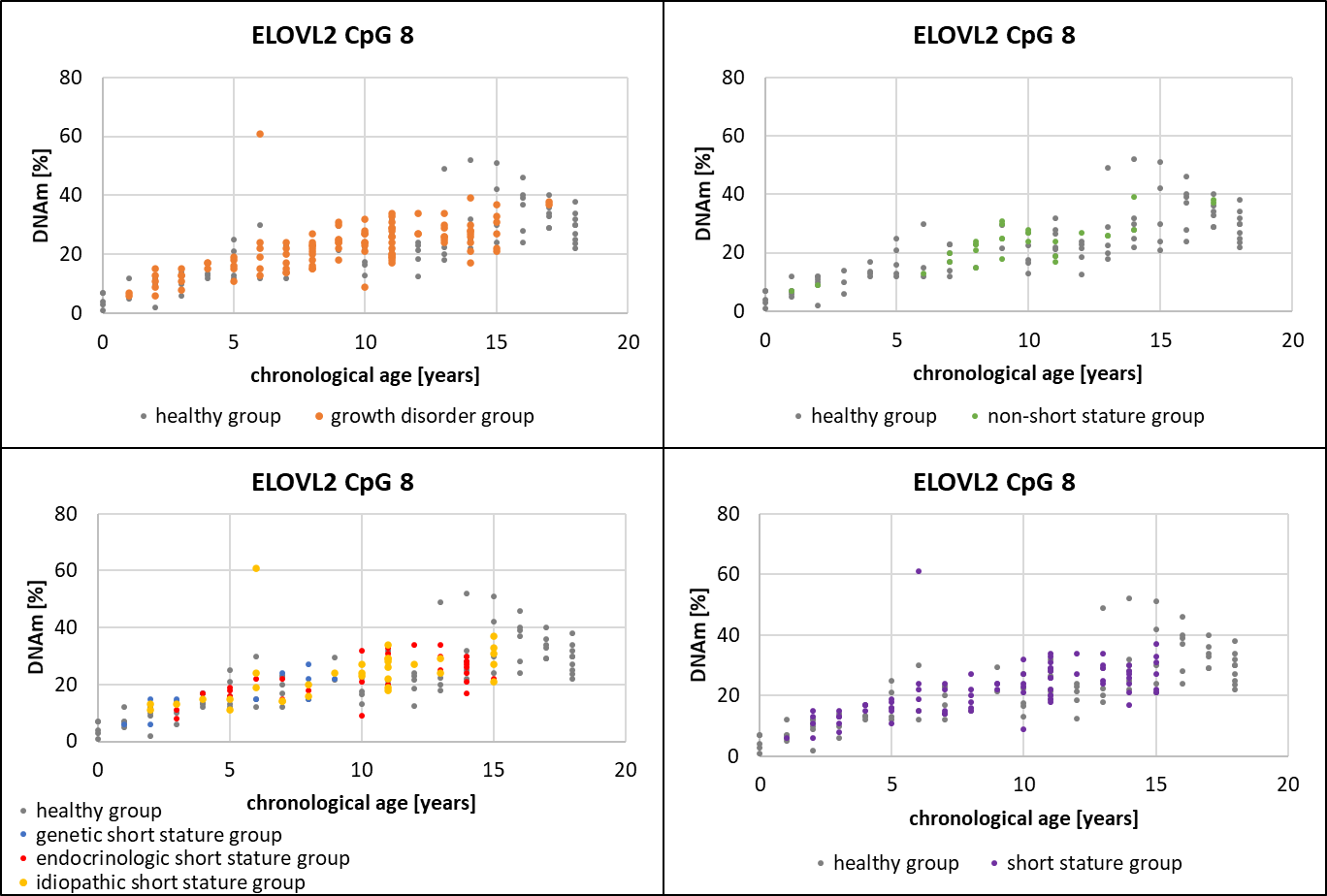


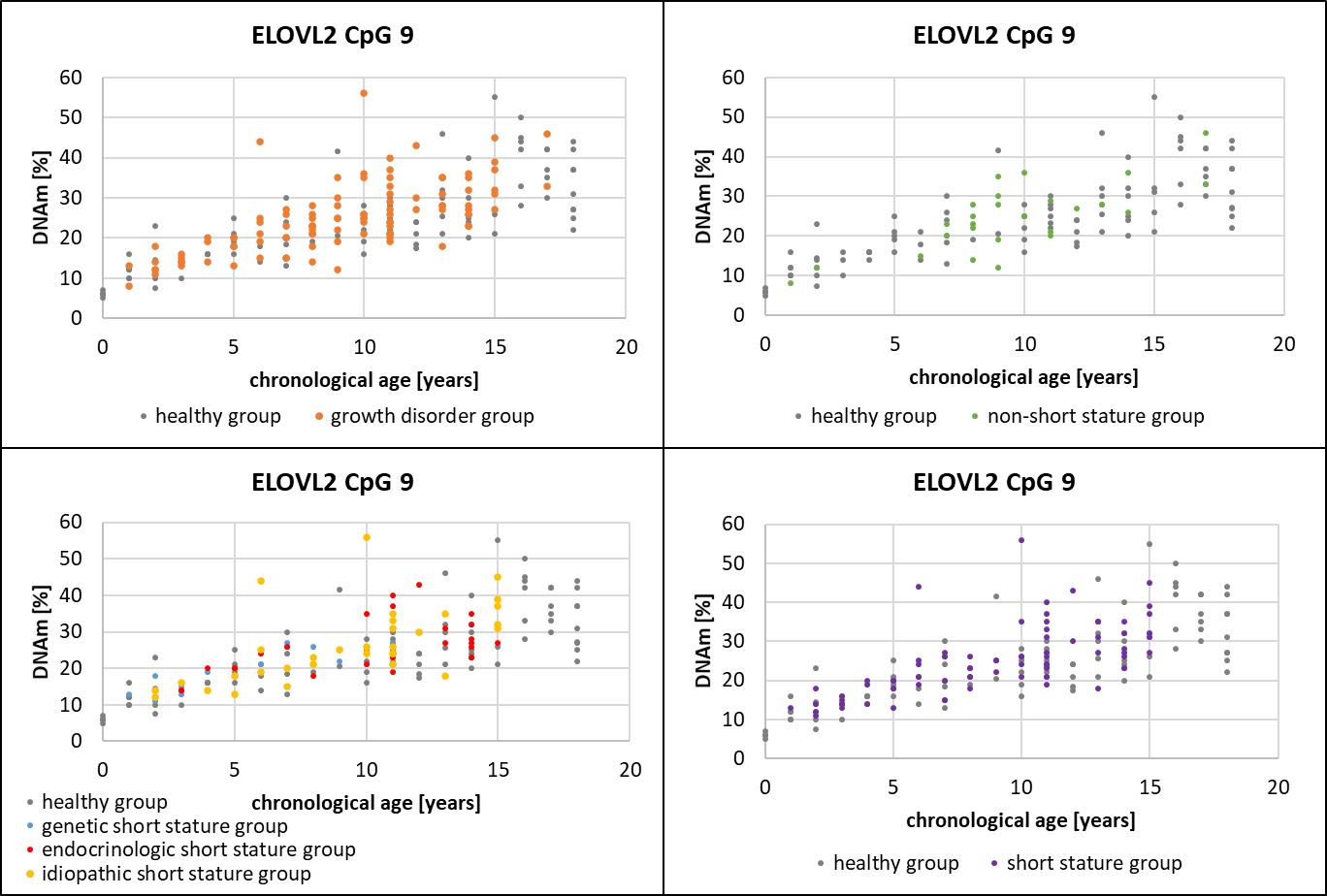


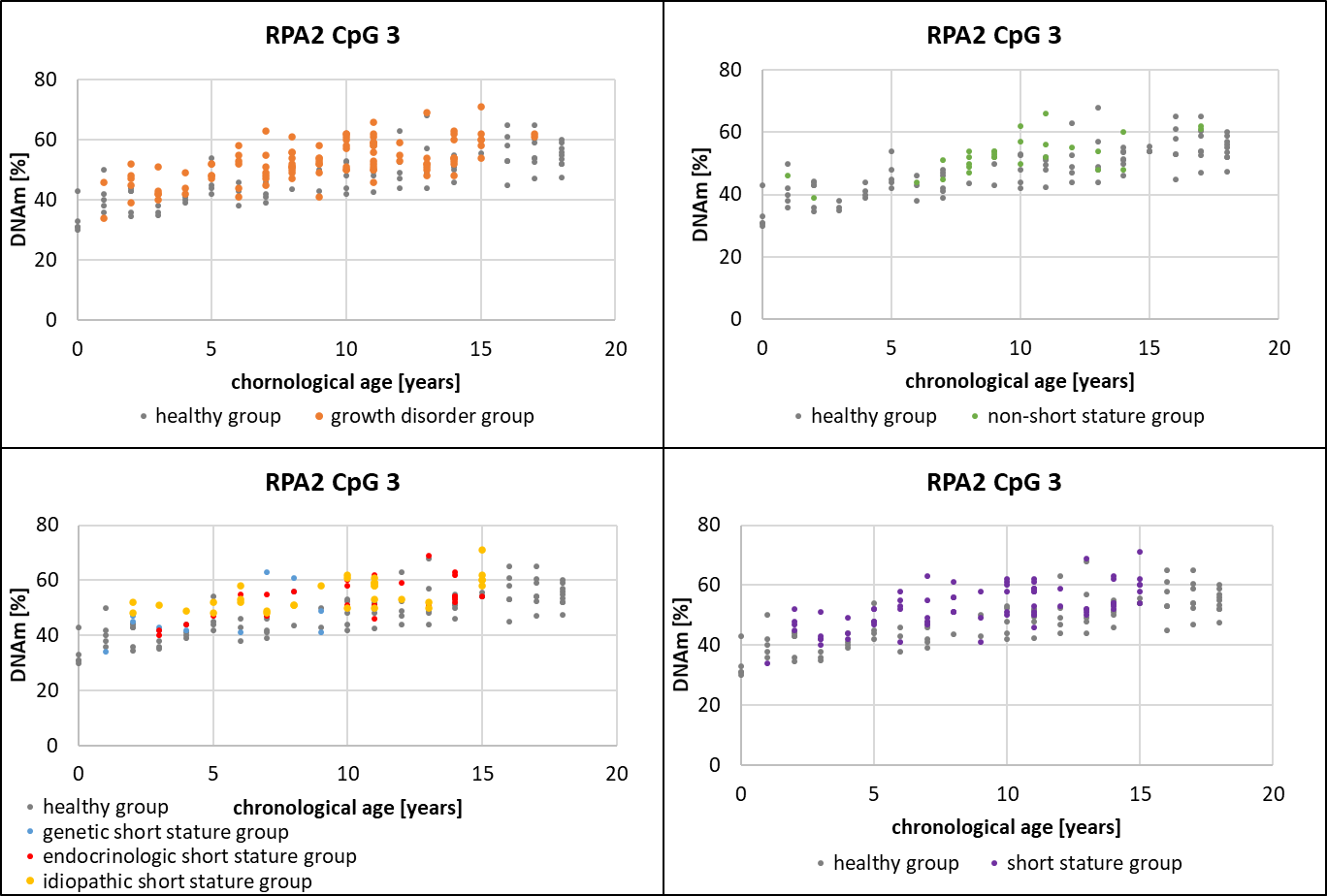


**Supplementary material figure 1**: Analysis results of DNAm vs age of sample donors for 11 CpG sites used for modelling.
